# Microplastic Release from Drinking Water Contact Materials: Material-Specific Responses to Chlorination and Implications for Consumer Exposure

**DOI:** 10.3390/molecules31213960

**Published:** 2026-11-03

**Authors:** Rodrigo D. M. Cordeiro, Ana Penetra, Rui N. Carneiro, Vitor V. Cardoso, Cristina M. M. Almeida

**Affiliations:** 1 Direção de Laboratórios, S.A.–EPAL, Empresa Portuguesa das Águas Livres, 1250-144 Lisboa, Portugal; rodrigo.cordeiro@adp.pt (R.D.M.C.); ana.penetra@adp.pt (A.P.); rui.carneiro@adp.pt (R.N.C.); vitor.cardoso@adp.pt (V.V.C.); 2 iMed.UL, Faculdade de Farmácia, Universidade de Lisboa, 1649-003 Lisboa, Portugal

**Keywords:** microplastics, drinking water, pipe materials, chlorination, migration tests, micro-FTIR

## Abstract

Microplastics are increasingly recognized as contaminants of emerging concern in drinking water, highlighting the need to assess drinking-water-contact materials as potential sources of particle release. This study investigated microplastic migration from six materials commonly used in drinking water infrastructure under chlorinated and non-chlorinated conditions using three consecutive migration cycles. Particle abundance, polymer composition, and size distribution were determined by micro-Fourier transform infrared spectroscopy (μ-FTIR). Microplastic release was strongly dependent on material composition. Polyester-derived particles predominated in M1 and M2, polyethylene particles in M3 and M4, and PVC particles in M5, whereas the cementitious lining material (M6) exhibited the lowest release potential. Chlorination influenced particle release in a material-dependent manner, with M2 showing the highest concentrations under chlorinated conditions. Across all materials, most particles belonged to the 20–50 μm and 50–100 μm size classes. Consumer exposure was assessed using a Relative Microplastic Release Index (RMRI), identifying M2 and M5 as the major contributors to potential exposure and M4 and M6 as the most stable materials. Overall, the results demonstrate that material composition governs microplastic release and should be considered when evaluating drinking-water-contact materials and consumer exposure.

## 1. Introduction

Ensuring the safety of water intended for human consumption has historically evolved from simple end-of-pipe monitoring at treatment plants to an integrated source-to-tap risk management paradigm. This modern approach, as set out in the recast Drinking Water Directive (EU) 2020/2184 [1], adopts a multi-barrier strategy, often compared to the Swiss cheese model in which multiple layers of protection, from catchment area protection to distribution network integrity, compensate for individual fail-safes. Within this framework, the distribution system and domestic installations have been identified as critical sources of secondary contamination [2]. Contact materials, while essential for infrastructure, may compromise water quality through physical degradation or chemical leaching, potentially undermining previous treatment steps [3,4]. As water utilities increasingly adopt polymer-based materials in place of traditional metallic infrastructure, understanding the potential for these materials to release microplastics (MPs) has become a critical component of drinking water safety assessment.

Among emerging contaminants of concern, MPs, polymeric particles smaller than 5 mm [5,6], have gained significant regulatory and scientific attention due to their ubiquity and potential impacts on public health [7]. Recent evidence indicates that MPs detected in tap water are not exclusively derived from environmental contamination of source waters but may also originate from the drinking water supply system itself through the degradation of materials used in distribution networks and household plumbing installations [8]. Modern drinking water distribution systems extensively employ polymers such as polyethylene (PE), polypropylene (PP), polyvinyl chloride (PVC), polyethylene terephthalate (PET), polyamide (PA), and cross-linked polyethylene (PEX) due to their durability, corrosion resistance, flexibility, and cost-effectiveness. These materials are composed of polymeric matrices with diverse, often complex molecular architectures [9,10]. Polymers are formed by the covalent bonding of monomeric units into linear, branched, or cross-linked macromolecular chains, yielding materials with distinct morphologies and physicochemical properties. Parameters such as molecular weight, crystallinity, chain branching, density, degree of cross-linking, and surface chemistry determine the mechanical performance and long-term stability of each polymer under operational conditions [11]. Although designed to ensure decades of service life, polymeric contact materials are continuously subjected to physical, chemical, and biological stressors within drinking water systems. Hydraulic forces, pressure fluctuations, temperature variations, flow conditions, disinfectant exposure, oxidative reactions, pipe age and interactions with dissolved constituents can progressively change the polymer structure and surface characteristics. These aging processes may induce chain scission, oxidation, surface embrittlement, cracking, abrasion, and fragmentation, ultimately resulting in the generation and release of MP particles into the water stream. The extent and nature of this release are strongly dependent on the intrinsic properties of the polymer matrix, with different materials exhibiting distinct degradation pathways and rates [12,13,14].

Consequently, the occurrence, abundance, morphology, and size distribution of MPs in drinking water are closely linked to the composition of the materials present throughout the supply infrastructure. Indeed, global studies consistently report that the polymer types most frequently identified in drinking water, including PE, PET, PP, PS, and PA, correspond not only to the most widely produced plastics worldwide, but also to those commonly used in water distribution systems and domestic plumbing installations [9,15]. As a result, the interaction between material-specific properties and environmental conditions ultimately governs both the generation of MPs and their fate within drinking water networks [14].

Beyond their occurrence, MPs in drinking water raise important public health concerns. Although current knowledge regarding the health effects of chronic exposure remains incomplete, growing evidence suggests that MPs may act not only as particulate contaminants but also as vectors for additives, residual monomers, and adsorbed environmental pollutants. Moreover, smaller particles, particularly those in the micro- and nanoscale range, may exhibit enhanced biological interactions and potentially cross physiological barriers, raising concerns about long-term human exposure through ingestion [7,16,17]. While substantial uncertainties remain regarding dose–response relationships and toxicological significance, the precautionary principle embedded in European drinking water legislation supports proactive efforts to identify and minimize avoidable sources of MP contamination in water supply systems.

This relationship between polymer characteristics, material aging, and microplastic release is particularly relevant within the regulatory framework established by the recast Drinking Water Directive (EU) 2020/2184 [1], which emphasizes preventive risk management across the entire supply chain. Since contact materials have been recognized as potential contributors to secondary contamination [4], evaluating their propensity to release MPs is essential to ensure that the integrity of the distribution system does not compromise the quality of water delivered to consumers. Such knowledge is also fundamental to supporting evidence-based material selection, improving infrastructure design, and strengthening public health protection at the point of consumption.

For this reason, analytical techniques capable of simultaneously detecting, quantifying, and chemically characterizing individual particles are required. In this regard, micro-Fourier Transform Infrared spectroscopy (µ-FTIR) has become one of the most widely adopted analytical tools for microplastic research. By combining microscopic imaging with infrared spectral analysis, µ-FTIR enables the identification of polymer types based on their characteristic vibrational fingerprints and provides information on particle size, morphology, and abundance [18]. This capability is particularly valuable in drinking water studies, where differentiating particles originating from distribution system materials from those introduced through source-water contamination is essential for understanding contamination pathways and supporting evidence-based material selection.

Therefore, the European Commission has established a harmonized methodology, as set out in Delegated Decision (EU) 2024/1441 [19], to standardize MP measurement across Member States and to prepare for their inclusion in the official drinking water watch list. For routine monitoring and precise identification, µ-FTIR has emerged as a high-throughput technique of choice because it enables the absolute counting and chemical characterization of particles ranging from 20 µm to 2000 µm, offering high resistance to interference from inorganic pigments and minerals commonly found in water matrices [20,21,22].

While the occurrence of MPs in drinking water has been widely reported, less attention has been paid to the release of particles directly from materials in contact with water and the corresponding implications for consumer exposure. Quantifying MP release and translating migration data into exposure estimates are important steps toward understanding the relative significance of different materials as emission sources. Furthermore, in the current absence of regulatory threshold values for microplastic release, comparative assessment tools based on exposure and relative release potential can help identify materials of greater concern, support evidence-based decision-making, and inform future risk assessment and regulatory frameworks.

Despite the growing body of literature on MP occurrence, experimental data directly correlating the specific chemical composition of contact materials with the characteristics of leached particles remain limited. This study aims to bridge this gap by experimentally analyzing six distinct plastic materials commonly utilized in water distribution systems. By employing µ-FTIR analysis, we seek to demonstrate a direct relationship between the polymer’s intrinsic properties and the count, size distribution, and morphology of microplastics leached into the water. The findings of this research are intended to provide essential data for selecting more inert materials, supporting the goals of the new European Positive Lists (EUPL) [23] and the overarching mandate for chemical safety at the source.

## 2. Results and Discussion

### 2.1. Characterization of Migration Waters

The following discussion about the target materials (Table 1) presents the migration behavior of each material individually, considering its composition, physical and chemical properties, and intended application. The discussion of the obtained results was based on (i) the dominant polymer species released, (ii) the effect of chlorination, (iii) changes observed over successive migration cycles, and (iv) the relationship between particle release and material characteristics. Table 1 and Tables S1–S3 (Supplementary Material) show the migration profile of these materials.

Based on the migration results and the characteristics of material M1 (TP1887 Polyester Resin, used as the GRP pipe matrix), the data suggest that polyester-derived particles are the dominant species released during migration, particularly in the absence of chlorine.

The migration profiles reveal notable differences between chlorinated and non-chlorinated conditions.

Under chlorinated conditions (M1), polyester particles remained the predominant polymer type detected, reaching approximately 56 particles/L during the third migration and 48 and 52 particles/L during the first and second migrations, respectively (Table S1). The observed increase in particle release under chlorinated conditions is consistent with the material’s greater susceptibility to chlorinated water exposure, although the underlying mechanisms were not directly investigated in this study. In addition, relatively low concentrations of polypropylene (PP), acrylonitrile–butadiene–styrene (ABS), polystyrene (PS), polymethyl methacrylate (PMMA), and other polymers were detected. The particle concentrations generally decreased with successive migration cycles, suggesting that chlorine may contribute to the oxidation and removal of loosely bound surface material during the initial exposures.

Under non-chlorinated conditions (M1), polyester release was substantially lower. Concentrations reached approximately 4 particles/L in the first migration, increased to 28 particles/L in the second migration, and then decreased to about 8 particles/L in the third migration (Table S1). These values indicate that chlorination appears to increase the release or persistence of polyester particles in suspension.

Polyester clearly dominates the particle composition in both exposure scenarios, which is consistent with the chemical composition of M1. The predominance of polyester particles is consistent with the resin matrix composition and suggests a material-related origin of the detected MPs.

ABS represents the second most abundant polymer, particularly under non-chlorinated conditions where concentrations ranged between approximately 4 and 12 particles/L. Lower levels of PP, PA, PS, EPDM, PMMA, and PVDF were also identified sporadically. These polymers are not major constituents of the polyester matrix and may originate from auxiliary materials, manufacturing processes, system components, analytical background contamination, or additives associated with the pipe production chain.

The temporary increase observed for polyester during the second migration under non-chlorinated conditions may indicate the continued degradation or detachment of surface resin fragments before stabilization occurs.

For M2 (CE66 Isophthalic Resin), the migration behavior differs markedly from M1 and reflects the higher hydrolytic resistance and lower water absorption of the isophthalic polyester resin. Compared with the orthophthalic polyester resin of M1, the isophthalic resin exhibits higher hydrolytic resistance, lower water absorption, greater flexibility and impact resistance, higher HDT (85 °C), and improved durability in aqueous environments [24]. These characteristics suggest a material expected to exhibit lower degradation rates and improved resistance to particle release during prolonged water contact.

Under chlorinated conditions, PA particles were the dominant polymer detected in all migration stages, increasing progressively from 148 particles/L (2nd migration) and 116 particles/L (third migration). This trend contrasts with M1, where particle concentrations tended to decrease over successive migrations.

ABS was the second most abundant polymer, with concentrations of approximately 36 particles/L (first migration), 16 particles/L (second migration) and 28 particles/L (third migration).

Small amounts of EPDM, PA, PS, PVC, PMMA and PP were also detected, generally below 12 particles/L.

In the absence of chlorine, polyester release was considerably high—80 particles/L (first migration), 112 particles/L (second migration), and 28 particles/L (third migration).

The peak observed during the second migration indicates a temporary release event, followed by stabilization in the third migration.

ABS was not detected under chlorinated conditions. Overall, particle release in non-chlorinated water was significantly higher relative to chlorinated conditions, especially in the second and third migrations.

The prevalence of polyester particles is consistent with the composition of M2, confirming that the isophthalic resin matrix is the main source of released MPs.

ABS and PA represent the other most abundant polymers. Since ABS and PA are not expected to constitute a major fraction of the resin matrix, its presence may be associated with manufacturing residues, auxiliary system components, pipe fittings or accessories, and background contamination during testing or analysis.

Other polymers (EPDM, PVC, PS, PMMA and PP) were detected only sporadically and at low concentrations.

The migration behavior of M3 (HDPE PE100) was characterized by the predominance of PE particles, confirming that the HDPE matrix is the principal source of released microplastics. Under chlorinated conditions, PE concentrations increased from approximately 48 particles/L during the first migration to 100 particles/L during the second migration, before decreasing sharply to about 8 particles/L in the third migration (Table S2). A similar trend was observed under non-chlorinated conditions, although PE concentrations remained higher throughout the test sequence. The pronounced reduction during the third migration suggests the exhaustion of readily releasable surface material and the stabilization of the pipe surface. The most important distinction is that M3 releases particles corresponding to its own polymer composition (PE), whereas M1 and M2 predominantly release polyester particles from the GRP resin matrix. Overall, the results indicate that HDPE PE100 is a relatively stable material, with particle release largely restricted to polyethylene fragments originating from the pipe surface and showing a tendency to decrease after repeated exposure cycles.

The migration behavior of M4 (polyethylene pipe) was characterized by the predominance of polyethylene particles under both chlorinated and non-chlorinated conditions, confirming that the pipe wall is the principal source of released MPs. The highest concentration was observed during the first migration in non-chlorinated water (approximately 112 particles/L), followed by a substantial decrease during subsequent migrations (Table S2). Under chlorinated conditions, PE concentrations decreased from approximately 92 to 76 particles/L between the first and second migration cycles and were no longer detected during the third cycle. These results indicate that particle release is largely associated with the removal of loosely attached surface material during the initial exposure period. The disappearance of detectable PE particles in the third migration suggests the stabilization of the pipe surface and limited long-term release potential. Overall, the KTubo polyethylene pipe exhibited an initial release behavior typical of polymeric materials, followed by a clear tendency towards stabilization under both exposure conditions.

The migration behavior of M5 (PVC-O) was dominated by PVC particles, confirming that the pipe wall material is the principal source of released MPs. Under chlorinated conditions, PVC concentrations decreased from approximately 56 particles/L during the first migration to 36 particles/L during the second migration and 28 particles/L during the third migration (Table S3). The observed decline under chlorinated conditions in particle release indicates the progressive depletion of readily releasable surface material and subsequent stabilization of the PVC-O surface. Lower concentrations were observed under non-chlorinated conditions, where PVC release remained approximately constant at 20 particles/L during the all the migration cycles. Compared with the GRP and HDPE materials, PVC-O showed moderate initial particle release but demonstrated rapid stabilization, suggesting good long-term resistance to microplastic generation under the tested conditions.

M6 is a cementitious lining (cement mortar), commonly used as an internal protective lining in water distribution systems. Unlike M1-M5, cement mortar is an inorganic material and not a polymeric matrix. Therefore, any polymer particles detected during migration tests are unlikely to originate directly from the cement mortar itself, and may instead result from contamination, auxiliary materials, or residues associated with sampling and analysis.

Consequently, the interpretation of the results should focus not only on the quantity of particles detected but also on the fact that the detected polymers are not constituents of the cementitious material.

The migration results obtained for M6 differ substantially from those observed for the polymeric materials investigated in this study. Under both chlorinated and non-chlorinated conditions, only low concentrations of sporadic polymer particles were detected, with maximum concentrations not exceedingly approximately 20 particles/L. Polyester and polystyrene were observed during the first migration under chlorinated conditions, whereas low levels of PP, PA PVC, and PTFE were detected under non-chlorinated conditions. Because these polymers are not constituents of cement mortar, the detected particles are unlikely to originate from the cementitious matrix itself and may instead reflect background contamination or ancillary materials associated with the experimental system. The complete absence of detectable particles during the third migration cycle indicates rapid stabilization and the absence of sustained particle release. Overall, M6 exhibited the lowest migration potential among all tested materials, highlighting the excellent stability of cement mortar with respect to microplastic generation under the investigated conditions.

The sporadic occurrence, low concentrations, and lack of consistent polymer patterns among migration cycles suggest that the detected particles are more likely associated with pre-existing surface contamination, handling, storage, transportation, or other secondary sources than with continuous microplastic release from the cementitious matrix itself. Moreover, the correspondence between material composition and released polymer types observed for M1-M5 was not observed for M6, further supporting a different origin for the particles detected in this material.

In conclusion, a general reduction in the number of MP particles released is observed by the third migration cycle in both test conditions for all materials. This behavior suggests an initial “wash-off” phase, during which particles weakly attached to the surface are removed during the first and second contact periods. After this stage, the system appears to approach a more stable release regime with lower particle emission rates (Tables S1–S3).

The Wilcoxon signed-rank test indicated that total MP concentrations were generally higher in chlorinated water (100.7 MPs/L) than in non-chlorinated water (60.2 MPs/L). However, the difference was not statistically significant at the 95% confidence level (W = 46, *p* = 0.090). This result suggests that the influence of chlorination is material-dependent, with some materials exhibiting enhanced particle release, whereas others remained largely unaffected. Consequently, material composition appears to be the dominant factor governing microplastic migration.

### 2.2. C_Tap_ Values

**Figure 1:**
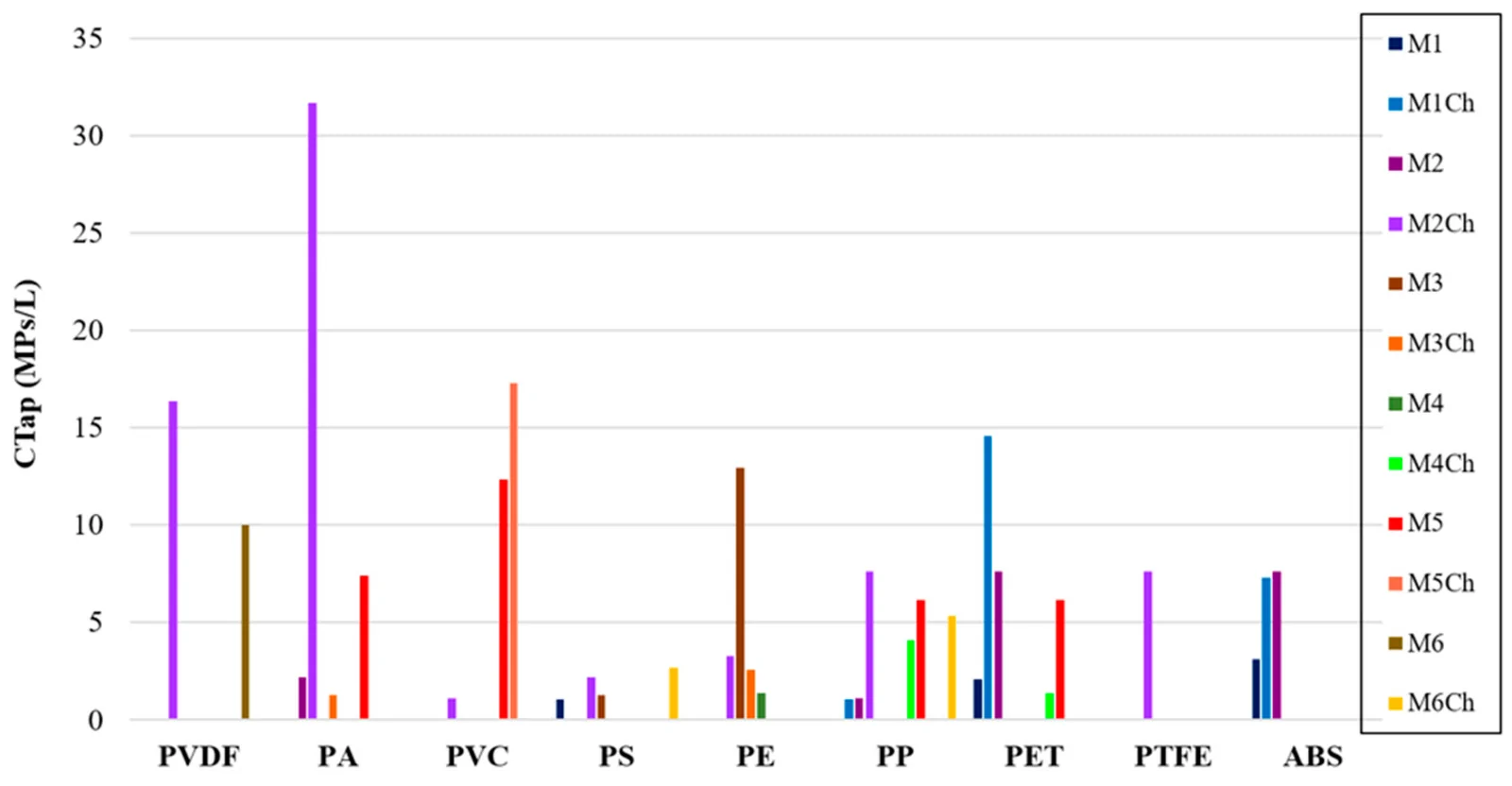
Comparison of *C_Tap_* concentrations (MPs/L) after the third migration cycle for materials M1-M6 exposed to demineralized water and chlorinated water (Ch).

The *C_Tap_* (MPs/L) results after the third migration revealed marked differences among the six investigated materials, and demonstrated that the migration behavior was strongly dependent on both the polymer type and the testing medium, as shown in Figure 1. Overall, chlorinated water enhanced *C_Tap_* release in most cases, although the magnitude of this effect varied considerably between materials.

The maximum *C_Tap_* concentration measured during the third migration was 31.7 MPs/L for PA in material M2 under chlorinated water, followed by 17.3 MPs/L for PVC in M5 under chlorinated water and 16.4 MPs/L for PVDF in M2 under demineralized water, highlighting the dominant influence of material composition and chlorination on particle release.

Material M2 exhibited the highest *C_Tap_* levels among all materials tested. In demineralized water, M2 released 16.4 MPs/L from PVDF, while substantially higher values were measured in chlorinated water, reaching 31.7 MPs/L for PA and 7.7 MPs/L for both PP and PTFE. These results indicate a pronounced sensitivity of M2 to chlorinated conditions, suggesting that oxidative processes may accelerate surface degradation and particle detachment.

M5 also showed elevated *C_Tap_* values, particularly for PVC, where concentrations increased from 12.3 MPs/L in demineralized water to 17.3 MPs/L in chlorinated water. Significant *C_Tap_* levels were additionally observed for PA (7.4 MPs/L), PP (6.2 MPs/L), and PET (6.2 MPs/L), indicating a broader tendency of this material to release particles across different polymer classes.

The tubular material M3 displayed a distinct behavior, with the highest *C_Tap_* concentration recorded for PE (12.9 MPs/L) under demineralized water conditions. Exposure to chlorinated water reduced the *C_Tap_* level to 2.59 MPs/L, whereas only minor releases were detected for PA (1.29 MPs/L). This behavior suggests that the mechanisms governing particle generation in M3 may differ from those observed for the other materials and are not necessarily intensified by chlorination.

For M1, particle release was generally low, except for PET and ABS. In PET, *C_Tap_* increased markedly from 2.1 MPs/L in demineralized water to 14.6 MPs/L in chlorinated water, representing one of the strongest chlorination effects observed in this study. Similarly, ABS concentrations increased from 3.1 to 7.3 MPs/L. These results suggest that chlorinated water promotes degradation processes leading to enhanced particle release from M1.

M4 exhibited comparatively low *C_Tap_* concentrations, with measurable values restricted mainly to PE (1.4 MPs/L), PP (4.1 MPs/L), and PET (1.4 MPs/L). Chlorinated water produced only minor increases, the largest being observed for PP (4.1 MPs/L), indicating a relatively stable behavior under the experimental conditions.

M6 was characterized by very limited particle release, with detectable *C_Tap_* concentrations only for PVDF (10.0 MPs/L) and PP (5.3 MPs/L) in demineralized water and PS (2.7 MPs/L) in chlorinated water. Compared with M2 and M5, M6 showed a substantially lower susceptibility to particle generation during repeated migration cycles.

Overall, the third migration results indicate that repeated exposure does not eliminate particle release, and that chlorinated water frequently promotes higher *C_Tap_* concentrations. The particularly high values recorded for M2 and M5 suggest that these materials are more vulnerable to degradation and particle emission, whereas M4 and M6 appear to exhibit greater stability during prolonged contact with water.

The absence of regulatory limits for microplastic release from drinking-water-contact materials prevents a compliance-based interpretation of *C_Tap_* values. Nevertheless, the results clearly differentiate the particle-release behavior of the investigated materials. In this context, lower *C_Tap_* values across successive migration cycles may be considered indicative of greater material stability, whereas elevated *C_Tap_* levels, particularly under chlorinated water exposure, suggest an increased susceptibility to degradation and particle generation. Consequently, *C_Tap_* can be regarded as a complementary performance metric that extends conventional chemical migration assessments by addressing the physical dimension of material deterioration.

### 2.3. Size Distribution of Released Microplastics

The MPs size (AED) distribution presented in this section (Table 2) corresponds to the third migration cycle, which was considered representative of stabilized particle release conditions following repeated water contact. The complete size distribution datasets for the third migration cycle under both chlorinated and non-chlorinated conditions are provided in the Supplementary Material (Table S4).

The size distribution of MPs detected after the third migration cycle revealed a clear predominance of small particles across all investigated materials. Most released particles were assigned to the 20–50 μm and 50–100 μm size classes, whereas particles larger than 300 μm were rarely detected. This pattern was observed under both chlorinated and non-chlorinated conditions, suggesting that the mechanisms responsible for particle generation preferentially produce fine microplastic fragments rather than larger particles.

For M1, M2, and M3, most particles were concentrated within the 20–100 μm range. Although some particles were identified in the 100–300 μm size class, their contribution was comparatively limited. Similarly, particles exceeding 300 μm were only sporadically detected, indicating that large-scale fragmentation was not a dominant degradation mechanism under the tested conditions.

Chlorination influenced particle abundance more markedly than particle size distribution. Despite the higher particle concentrations observed for some materials under chlorinated conditions, the dominant size classes remained largely unchanged. For example, M2 exhibited increased particle release in chlorinated water, but the released particles were still predominantly distributed within the 20–50 μm and 50–100 μm classes. Similar behavior was observed for M1 and M5, suggesting that chlorination primarily enhanced particle generation rather than altering the fragmentation pattern itself.

The predominance of particles smaller than 100 μm is particularly relevant from an exposure perspective, as these particles remain more readily suspended in water and are therefore more likely to reach the consumer tap. The consistent dimensional profiles observed among materials further suggest that particle size is governed mainly by the degradation and fragmentation processes occurring at the material surface, whereas the magnitude of particle release is more strongly influenced by material composition and susceptibility to oxidative degradation.

It should be noted that the reported size distribution refers exclusively to particles ≥ 20 µm, which was the lower quantification threshold adopted in this study. Therefore, particles smaller than 20 µm were not included in the assessment. The predominance of the 20–50 µm and 50–100 µm size classes should consequently be interpreted within the analytical range for which robust particle identification and quantification could be achieved using µ-FTIR.

### 2.4. Relative Microplastic Release Index (RMRI)

To evaluate the implications of microplastic release for consumer exposure and to facilitate comparison among materials, the sum of *C_Tap_* values of all identified polymers (Σ*C_Tap_*) was converted into Estimated Daily Intake (EDI), Annual Estimated Intake (AEI), and Relative Microplastic Release Index (RMRI) values. While EDI and AEI provide estimates of potential exposure through drinking water consumption, the RMRI functions as a comparative indicator of material performance, allowing the identification of materials with higher or lower microplastic release potential.

The RMRI was developed as a relative release indicator designed to compare the performances of different materials under standardized migration conditions. It does not represent a toxicological risk metric and should not be interpreted as an indicator of health risk to consumers.

Consequently, higher RMRI values indicate a greater relative contribution to consumer exposure, whereas lower values suggest improved material stability during water contact. The calculated exposure metrics and RMRI values for chlorinated and non-chlorinated water are summarized in Table 3.

Under non-chlorinated conditions, the highest RMRI was observed for M5 (1.00), followed by M2 (0.58) and M3 (0.44), indicating that these materials contributed most strongly to the estimated annual intake of microplastics. In contrast, M4 (0.04) and M6 (0.00) exhibited the lowest RMRI values, suggesting minimal particle release under the investigated conditions. These results demonstrate substantial differences in release behavior among materials, and indicate that material composition and manufacturing characteristics play a major role in governing microplastic generation.

A markedly different pattern was observed under chlorinated conditions. M2 exhibited the highest RMRI value (1.00), corresponding to an estimated annual intake of 51,027 particles year^−1^, while all remaining materials showed considerably lower indices. M1 (0.33) and M5 (0.25) formed an intermediate group, whereas M3 (0.06), M4 (0.08), and M6 (0.11) remained characterized by relatively low release potential. The increase observed for M2 suggests that this material is particularly susceptible to particle generation under oxidative conditions, whereas the comparatively low RMRI values obtained for M3 and M4 indicate greater resistance to disinfectant-induced degradation.

From a material performance perspective, the RMRI provides a useful framework for differentiating materials according to their long-term release behavior. M4 consistently exhibited low RMRI values under both water matrices. M6 presented an RMRI value of 0.00 under non-chlorinated conditions and 0.11 under chlorinated conditions, remaining within the low-release-potential category defined in this study. These results suggest that both materials exhibited comparatively low microplastic release relative to the other materials investigated. Conversely, M2 consistently ranked among the highest contributors to potential consumer exposure, indicating that this material warrants particular attention in future studies addressing the mechanisms of particle generation and long-term aging.

A one-way ANOVA indicated that chlorination did not significantly affect RMRI values (*p* > 0.05). However, differences in material ranking were observed between chlorinated and non-chlorinated conditions, suggesting that the response to chlorination is material-dependent. Although these differences were not statistically significant, the RMRI results indicate that materials contribute differently to potential MP exposure and provide a useful basis for ranking materials according to their relative release potential. Consequently, RMRI may serve as a complementary tool for material selection and prioritization within drinking water distribution systems.

### 2.5. Implications for Drinking Water Infrastructure

The results obtained in this study have direct implications for the selection, operation, and long-term management of drinking water distribution infrastructure. The observed differences in MP release among the investigated materials demonstrate that particle generation is not exclusively governed by the polymer family itself, but also by formulation characteristics, additives, manufacturing processes, and material aging resistance. This finding highlights the importance of considering MP release as an additional performance criterion when evaluating materials intended for contact with drinking water, particularly in the context of increasingly stringent water quality requirements.

From an operational perspective, the results indicate that materials M4 and M6 exhibited the lowest *C_Tap_* values and the most stable behavior throughout the migration assays, suggesting a lower propensity for long-term particle release. In contrast, M2 and M5 consistently generated higher *C_Tap_* values, especially under chlorinated conditions, indicating a greater susceptibility to particle detachment and surface degradation. Although all materials complied with current regulatory requirements governing drinking-water-contact products, the marked differences in MP release demonstrate that regulatory compliance alone may not fully capture the physical stability of materials during service. Consequently, incorporating particle release assessments into material qualification procedures could provide valuable complementary information for utilities and infrastructure managers.

The influence of chlorination observed for several materials is particularly relevant because disinfectant residuals are routinely maintained throughout drinking water networks to ensure microbiological safety. The increased particle release detected for some materials under chlorinated conditions suggests that oxidative aging mechanisms may contribute to MP generation during normal operation. As distribution systems are expected to remain in service for several decades, even relatively low particle release rates may result in continuous microplastic inputs throughout the asset lifetime. Therefore, future material selection strategies should consider not only traditional criteria such as mechanical performance, corrosion resistance, hydraulic efficiency, and chemical migration, but also the propensity for microplastic release under realistic operating conditions.

It should be noted that the migration tests were performed under standardized laboratory conditions using demineralized water and a fixed initial free chlorine concentration. Therefore, the results are intended to represent a controlled migration scenario and may not fully reflect the complexity of operational drinking water systems, where factors such as pH, alkalinity, natural organic matter, ionic composition, and chlorine decay may influence material behavior and particle release.

Integrating particle release evaluation into drinking water risk assessment frameworks could support the development of more sustainable and resilient distribution systems while reducing consumer exposure to MPs.

These findings support the inclusion of microplastic release testing as a complementary parameter within future European assessment frameworks for drinking-water-contact materials, alongside conventional chemical migration and toxicological evaluations.

## 3. Materials and Methods

### 3.1. Reagents and Materials

Migration studies were performed with demineralized water obtained by reverse osmosis, Elga Centra R200 (Veolia, Aubervilliers, France).

Free chlorine stock solutions were prepared by dilution from purchased 12% hypochlorite solution (Sigma-Aldrich, St. Louis, MO, USA). Intermediate and standard solutions were prepared from sodium hypochlorite stock solutions to obtain chlorine solutions at 10 mg/L. Before analysis, the water samples were spiked to obtain water solutions at 1 mg/L.

The filtration process was carried out on a vacuum filtration system (KGS-04, Advantec Co., Ltd., Tokyo, Japan) with a silicon filter of 1 cm^2^, an effective filtration area of approximately 0.66 cm^2^, and pores of 5 μm (SmartMembranes GmbH, Halle (Saale), Germany). Blanks and filtered samples were carried to the micro-FTIR laboratory in closed borosilicate glass Petri dishes (Ø 25 mm).

### 3.2. Material Samples

Table 4 and Table 5 show the main physical, chemical, thermal, and functional characteristics of the materials employed in the water supply system, as well as dimensions, *S*/*V* ratio and conversion factors, respectively. Data were obtained from manufacturers’ technical datasheets, certification documents, and potable water compliance reports.

Thermoset resins (M1-M2), thermoplastic pipes (M3-M5), and cementitious linings (M6) were tested under exposure conditions representative of their service environments, following the principles established under the European framework for products in contact with drinking water [1,26].

Materials M1, M2, M5, and M6 were supplied as test specimens, whereas materials M3 and M4 were provided as tubes. The received geometries were considered during specimen preparation and experimental testing.

These target materials (M1-M6) used in the water supply system have been selected and subjected to migration tests. The amount of material used for each test depends on the surface-to-volume ratio (*S*/*V*) of each material. The *S/V* ratio adopted for migration testing varied according to the material type, geometry, and intended application in the water distribution system. Therefore, this study adopted several *S/V* ratios according to the target material. The *S/V* ratio was adjusted according to the material type and intended application within the water distribution system.

### 3.3. Migration Assays

**Figure 2:**
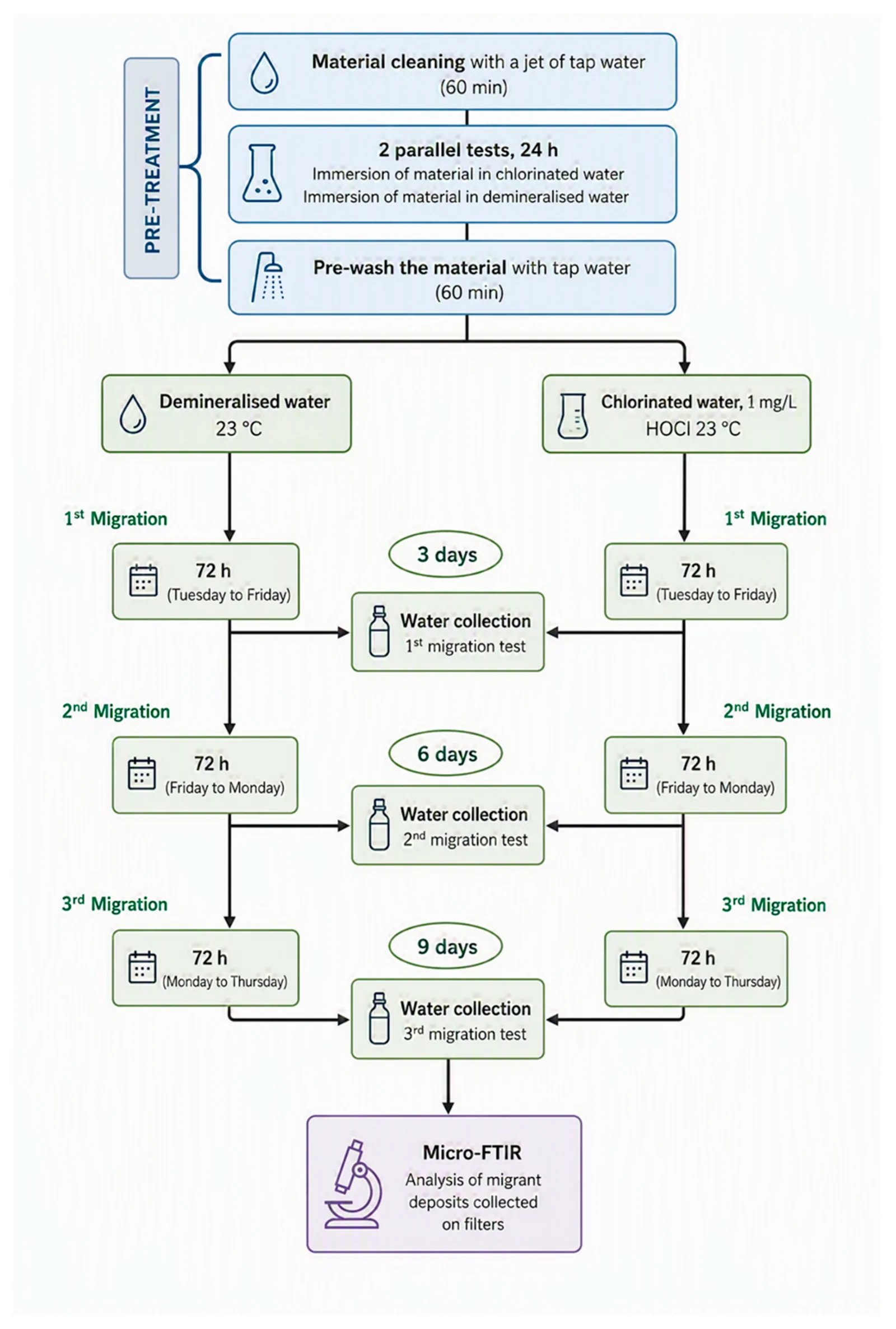
Material migration assay for identification and quantification of microplastics by µ-FTIR.

The migration tests, which follow the requirements of EN 12873-1 [27], were carried out over 10 days using demineralized water and chlorinated water (1 mg/L of chlorine). A test blank with demineralized water was also performed. The migration tests included 1 day of pre-treatment and 9 days of the procedure, followed by a µ-FTIR analysis of MPs (Figure 2).

These migration tests were conducted to evaluate the potential release of MP particles under different water quality conditions. For each material, three consecutive migration cycles were performed, allowing the assessment of both the initial particle release and the evolution of migration behavior over repeated water contact periods.

The tests included a pre-treatment phase consisting of cleaning the materials with a jet of tap water (TW) for 60 min, followed by immersion in demineralized water (DW) for 24 h. After this treatment, the immersion water was discarded, and the materials were again cleaned with a jet of TW for 60 min. After pre-treatment, the migration test involved immersing the materials in the test water at 23 °C for 72 h. At the end of these three days, the migration water was collected and analyzed by µ-FTIR (1st migration). A further two migration procedures were carried out at 3-day intervals, after which the demineralized water was replaced [27].

At the end of each 72 h migration cycle, the test water was discarded and replaced with freshly prepared migration water prior to the subsequent migration stage. For chlorinated water experiments, a new chlorine solution was prepared and adjusted to an initial free chlorine concentration of 1 mg/L at the beginning of each migration cycle. Accordingly, the reported chlorine concentration corresponds to the initial concentration applied during each migration period.

The concentration of the MPs in the migration waters was obtained with Equation (1), where $c_{n}^{T}$ is the concentration of the MP particles leached from the material expressed in number of MP particles per liter (or MPs/L), $a_{n}^{T}$ is the concentration of the MPs expressed in MPs/L measured in the migration water, and $b_{n}^{T}$ is the concentration of the MPs expressed in MPs/L measured in the blank test.

(1) $$c_{n}^{T}=a_{n}^{T}-b_{n}^{T}$$

The migration rate of a given MP particle ($M_{n}^{T}$—measured in MP dm^−2^ d^−1^) in migration water is calculated from its concentration in that water, according to Equation (2), where *t* is the period of the migration test, expressed in days (3 days in this test), and *S*/*V* is the surface area/volume ratio, expressed in dm^−1^.

(2) $$M_{n}^{T}=\frac{c_{n}^{T}}{t\times S/V}$$

Different *S*/*V* ratios were used for M1-M6 to reflect their intended end-use conditions and exposure configurations (Table 5). The selected ratios followed the requirements and testing principles established in Commission Delegated Regulation (EU) 2024/370 [26] and the harmonized testing methods adopted under Directive (EU) 2020/2184 [1] for materials in contact with drinking water.

The estimated concentration at the consumer tap (*C_Tap_*) was determined using the migration rate from the third migration test and the conversion factor (*CF*) of each material, according to Equation (3).

(3) $$C_{\mathrm{Tap}}=M_{n}^{T}\times \mathrm{CF}$$

To estimate consumer exposure under realistic operating conditions, material-specific *CFs* were adopted instead of a single universal value. This approach accounts for differences in the distribution, geometry, and exposure conditions of each material within the water distribution network [28]. This is consistent with the European regulatory framework for products intended to come into contact with drinking water, which requires assessment under foreseeable conditions of use.

The selected *CF* values were based on the intended application and representative pipe diameters of each material. Materials associated with transmission mains and large-diameter pipes (M1, M2 and M6) were assigned a *CF* of 5, corresponding to pipes with internal diameters ≥ 300 mm. Thermoplastic materials typically used in service and distribution pipes (M3, M4 and M5) were assigned a *CF* of 10, representative of pipes with internal diameters between 80 and 300 mm. This approach reflects the expected hydraulic relevance and wetted surface-to-volume ratio of each material within the drinking water distribution network [29].

The adopted *S*/*V* ratios and *CF* were selected according to the intended application of each material and follow the requirements established in the European regulatory framework for drinking-water-contact materials [29]. These parameters account for differences in geometry, hydraulic conditions, wetted surface area, and expected human exposure. Therefore, the use of material-specific *S/V* ratios and *CF* provides representative estimates of migration and exposure under realistic use conditions, and ensures consistency with regulatory assessment procedures.

Consequently, comparisons among materials were not based on identical geometrical exposure conditions, but on application-specific conditions considered representative of their intended use within drinking water systems.

For organic compounds, consumer exposure was evaluated by comparing the estimated concentration at the tap (*C_Tap_*) with the maximum tolerable concentration at the tap (*MTC_Tap_*). Acceptance criteria were met when *C_Tap_* did not exceed *MTC_Tap_* after the third migration period (10 days), or after the ninth migration period (31 days) when extended testing was performed [29]. For the identified substances, the applicable *MTC_Tap_* corresponded to the substance-specific threshold value. In the absence of such a value, a default limit of 1.0 μg/L was applied. For unidentified substances, the threshold was set at 1.0 μg/L per compound and 5.0 μg/L for the sum of all unknown compounds [29].

For MP particles, *C_Tap_* is expressed as the number concentration of released particles (MPs/L). Therefore, the results cannot be directly assessed against the *MTC_Tap_* values established in Decision (EU) 2024/367 [23], which are defined for chemical substances and expressed as mass concentration (μg/L). Therefore, the *C_Tap_* results were evaluated comparatively to identify differences in particle-release behavior among materials, polymer types, migration periods, and water qualities. Higher *C_Tap_* values were interpreted as indicative of greater susceptibility to particle generation and release during water contact. Particular attention was paid to the influence of chlorinated water and repeated migration cycles on the persistence of particle release, as these parameters provide insight into the long-term stability of drinking-water-contact materials.

The comparison between chlorinated and non-chlorinated conditions provides insight into the potential influence of disinfectants on material degradation and particle release. Furthermore, identifying the polymer types released enables the assessment of whether the detected particles originate from the tested material itself or from secondary sources such as additives, auxiliary components, manufacturing residues, or background contamination.

Migration experiments were performed in duplicate (n = 2). Replicates corresponded to two independent test specimens of each material subjected to separate migration assays under identical experimental conditions. The three migration cycles were conducted sequentially on each specimen following the procedure described in EN 12873 [27]. The reported values correspond to the mean concentration obtained from the duplicate migration experiments.

### 3.4. Leachate Collection

Leachates obtained from materials intended for contact with demineralized water and chlorinated water were analyzed for microplastic release. Leachates were collected in pre-cleaned amber glass containers for microplastic analysis. Samples were stored at 2–8 °C and analyzed within seven days. All handling procedures were conducted under contamination-controlled conditions, avoiding the use of plastic laboratory materials whenever possible.

### 3.5. Sample Preparation and µ-FTIR Analysis

Microplastic identification and quantification were performed according to the µ-FTIR methodology previously developed and validated by Cordeiro et al. (2025) for aqueous matrices [9]. Briefly, 250 mL aliquots of leachates were vacuum-filtered through 5 μm pore-size silicon filters (SmartMembranes GmbH, Halle (Saale), Germany), previously identified as the optimum compromise between particle retention, filter overloading, and analytical performance. Following filtration, filters were transferred to borosilicate glass Petri dishes and stored until spectroscopic analysis. No sample digestion, density separation, or chemical pretreatment was applied.

Microplastic particles were identified and quantified using a Nicolet™ N10 micro-Fourier Transform Infrared (µ-FTIR) microscope (Thermo Fisher Scientific, Waltham, MA, USA), equipped with a liquid nitrogen-cooled mercury cadmium telluride (MCT) detector and operated in transmission mode. Spectra were acquired within the 4000–675 cm^−1^ spectral range at a resolution of 8 cm^−1^ using 16 scans per spectrum. Analyses were carried out using a 100 μm × 100 μm aperture, previously optimized during method validation. A background spectrum was collected from a clean silicon filter before each analytical sequence [9].

The entire effective filter area was analyzed in order to avoid the application of area-correction factors and to minimize uncertainty associated with the non-uniform distribution of particles on the filter surface. Full-filter analysis was considered particularly important for samples containing low particle abundances [9].

The spectral comparison between the samples and the reference libraries was carried out in two polymer-specific regions, 3500–2700 cm^−1^ and 1800–700 cm^−1^. These ranges were selected because the typical FTIR spectra of MPs exhibit characteristic bands, including C=O stretching in 1700–1790 cm^−1^, C–H stretching at 2980–2780 cm^−1^, and C–H bending together with aromatic ring stretching at 1480–1430 cm^−1^. These functional groups are essential for determining the types of MP particles present in water samples [30,31,32].

Particle detection and polymer identification were performed using the OMNIC™ Picta™ software package, version 1.8.240 (Thermo Fisher Scientific, Waltham, MA, USA). Spectral matching was carried out using commercial polymer libraries supplied by Thermo Fisher Scientific and an in-house spectral database developed and validated by our research group. Only spectra presenting a hit quality index (HQI) of at least 65% were considered acceptable for polymer identification [9]. Spectral matching was performed using both commercial reference libraries and an in-house polymer library previously developed and validated by the authors. Spectra with HQI values below 65% were not considered for polymer identification, thereby reducing the likelihood of false-positive assignments.

The dimensions (length and width) of individual particles were automatically determined by the software during particle analysis. Some regulatory documents adopt different ways of quantifying particle size, namely, the area equivalent diameter (*AED*) [19], which was used for grouping the MP particles by their size.

Although the µ-FTIR method is capable of detecting particles down to approximately 10 µm, only particles with an equivalent diameter ≥ 20 µm were quantified in the present study. This threshold was adopted because it corresponds to the size range in which µ-FTIR analysis in transmission mode provides sufficient analytical robustness for reliable particle quantification and polymer identification [6,9,30].

### 3.6. Method Performance

The validation studies demonstrated an average recovery of 91%, with a relative standard deviation of 13%. Detailed information regarding method optimization, validation procedures, analytical figures of merit, and the development of the in-house polymer spectral library has been previously reported by Cordeiro et al. [9].

Daily quality assurance and quality control procedures included the use of procedural blanks and contamination prevention measures throughout sampling and analysis.

Procedural blanks were performed in parallel with all migration experiments using demineralized water and chlorinated water under identical experimental conditions but in the absence of test materials. These blanks were used to assess potential contamination arising from water quality, sample handling, laboratory conditions, filtration procedures, glassware cleaning, and analytical processing. Blank concentrations were determined for each migration condition and subtracted from the corresponding migration water concentrations according to Equation (1). The reported concentrations therefore represent blank-corrected values attributable to material-associated particle release.

### 3.7. Exposure Assessment and Relative Release Risk Index

As no regulatory threshold values are currently available for MP particle release from drinking-water-contact materials, a comparative exposure assessment approach was adopted. Consumer exposure was estimated using the concentration at the tap (*C_Tap_* ), calculated from the migration rate obtained during the third migration cycle and the corresponding material-specific conversion factor (*CF*).

The third migration was selected in accordance with the regulatory approach used for the migration testing of drinking-water-contact materials, and was considered more representative of post-conditioning release behavior following the initial migration periods. This migration stage was used as a standardized basis for comparison among materials, and should not be interpreted as evidence of long-term material stability.

The estimated daily intake (*EDI*) of microplastics was calculated according to Equation (4), assuming a daily drinking water consumption of 2 L per person,

*EDI* = *C_Tap_* × *IR* (4)

where *EDI* is the estimated daily intake (MPs/d), *C_Tap_* is the particle concentration at the consumer’s tap (MPs/L), and *IR* is the daily water ingestion rate (L/d).

The annual estimated intake (AEI) was determined according to Equation (5),

*AEI* = *EDI* × 365 (5)

where *AEI* represents the estimated annual MP intake expressed as particles per year (MPs/y).

The EDI and AEI calculations are based on standardized laboratory migration data and simplified exposure assumptions. Therefore, these values should not be interpreted as estimates of actual consumer exposure, but rather as comparative indicators intended to facilitate the evaluation and ranking of materials according to their relative microplastic release potential.

To compare the relative contribution of the investigated materials to potential consumer exposure, a Relative Microplastic Release Index (*RMRI*) was calculated based on the estimated annual intake (*AEI*). The *RMRI* was defined as the ratio between the annual intake associated with a given material and the highest annual intake observed among all tested materials, according to Equation (6),

(6) $$\mathrm{RMRI}=\frac{\mathrm{AE}I_{i}}{\mathrm{AE}I_{\max}}$$

where *RMRI* is the relative microplastic release index for material *i*, *AEI*_1_ is the annual estimated intake associated with the material under evaluation (MPs/y), and *AEImax* is the maximum annual estimated intake observed in the study (MPs/y).

The *RMRI* is a dimensionless parameter ranging from 0 to 1, with values closer to 1 indicating a greater relative contribution to potential consumer exposure through drinking water.

For interpretation purposes, materials were classified into four categories: low release potential (*RMRI* < 0.25), moderate release potential (0.25 ≤ *RMRI* < 0.50), high release potential (0.50 ≤ *RMRI* < 0.75), and very high release potential (*RMRI* ≥ 0.75).

The classification thresholds were established a priori by dividing the normalized RMRI scale (0–1) into four equal intervals to facilitate data interpretation.

Since no health-based guideline values are currently available for MP concentrations in drinking water [22,33,34], the *EDI*, *AEI*, and *RMRI* calculations were not intended as toxicological risk assessments. Instead, these metrics were used to compare the relative performance of the investigated materials regarding microplastic release and potential consumer exposure under the tested conditions.

Statistical differences between *RMRI* values obtained under chlorinated and non-chlorinated conditions were evaluated using a one-way analysis of variance (ANOVA), which is mathematically equivalent to an independent samples *t*-test when only two groups are compared.

Due to the limited number of independent experimental replicates inherent to standardized migration testing, the statistical analyses were considered exploratory and were used primarily to support the interpretation of observed trends, rather than to establish definitive inferential conclusions.

## 4. Conclusions

This study demonstrates that the composition of drinking-water-contact materials is the primary factor governing MP release under realistic migration conditions. For most investigated materials, the polymer types identified in the migration waters corresponded closely to the composition of the original material, supporting a material-related origin of the detected particles. These findings suggest an association between drinking-water-contact materials and the polymer composition of microplastics detected in migration waters.

Differences in MP release were observed among materials, and the effects of chlorination varied according to the material investigated. Materials M2 and M5 consistently exhibited the highest *C_Tap_*, *AEI*, and *RMRI* values, whereas M4 and M6 showed the lowest particle release potential under the experimental conditions investigated, and exhibited lower particle release during the later migration stages.

The reduction observed during successive migration cycles suggests a tendency towards lower particle release under the tested conditions; however, the present study was not designed to assess long-term material stability over the full service lives of the materials.

Although chlorination did not produce statistically significant differences in RMRI values, differences in particle release were observed between chlorinated and non-chlorinated conditions for several materials. These observations suggest that chlorinated water may influence migration behavior; however, the present study was not designed to identify the underlying mechanisms responsible for the observed differences.

Across all investigated materials, most released particles belonged to the 20–50 μm and 50–100 μm size classes, while particles larger than 300 μm were rarely observed. This consistent size distribution indicates that the released particles were predominantly present as fine fragments within the analytical range investigated. It should be noted that the reported size distribution corresponds to particles ≥ 20 µm, which was the lower quantification limit adopted in this study. The predominance of small particles, combined with the exposure estimates obtained through *C_Tap_*, *EDI*, *AEI*, and *RMRI* calculations, highlights the relevance of MP release as a complementary indicator of material performance. The *EDI*, *AEI*, and *RMRI* metrics were used exclusively as comparative tools derived from standardized laboratory migration data, and were not intended to represent actual consumer exposure or toxicological risk.

The present results demonstrate an association between material composition and the polymer composition of released particles. However, the study was not designed to determine the physicochemical mechanisms responsible for particle release, which should be addressed in future studies using complementary surface characterization techniques.

Overall, the results demonstrate that conventional chemical migration assessments alone may not fully characterize the long-term behavior of drinking-water-contact materials. The integration of MP release testing, particle characterization, and exposure-based indicators provides complementary information that may support the evaluation and selection of drinking-water-contact materials.

## Figures and Tables

**Table 1:** Migration behavior of each material with chlorinated water and non-chlorinated water (*n* = 2).

	Chlorinated Water					
	Maximum Concentration (MPs/L)	Migration Cycle	Dominant Polymer	Other Polymer (MPs/L)		
				1st Migration	2nd Migration	3rd Migration
M1	56	3rd	PET	ABS (44), PMMA (4)	ABS (20), PP (4), PA (12), PS (8)	ABS (28), PP (4)
M2	148	2nd	PA	PVDF (56), ABS (44)	PS (120), PE (120), PTFE (68), PP (60), EPDM (4), ABS (20)	PA (116), PVDF (60), PTFE (28), PP (28), ABS (28), PE (12), PVC (4), PS (8)
M3	100	2nd	PE	PET (64), PP (8)	PET (16), PP (12)	PA (4)
M4	92	1st	PE	PA (8), PET (16), PP (4)	PVC (4), PET (8), PP (4)	PET (4), PP (12)
M5	56	1st	PVC	PA (4), PS (8)	PC (4), PET (4)	PA (12)
M6	20	1st	PET	PS (8), PE (8)	PE (4)	PP (8), PS (4)
	Non-Chlorinated Water					
M1	28	2nd	PET	EPDM (4), PA (4), ABS (4)	PS (12), ABS (12)	PS (4), PET (8), ABS (12)
M2	112	2nd	PET	PVDF (4), PA (8), PMMA (4), PP (8), ABS (36)	EPDM (8), PA (8), PS (12), ABS (16)	PA (8), PMMA (0), PP (4), ABS (28)
M3	80	2nd	PE	PET (8), PP (16)	PA (4), PET (16), PP (8), PS (8)	PS (4)
M4	112	1st	PE	EPDM (12), PA (8), PVC (12), PP (12), PET (8), PTFE (4)	PTFE (4)	None detected
M5	20	1st–3rd	PVC	None detected	PP (10), PET (10)	PA (12), PP (10), PET (10)
M6	20	1st	PET	PE (12), PP (12), PTFE (4)	PA (4), PVC (4), PP (4)	None detected

**Table 2:** Size distribution of microplastics released during the third migration cycle from drinking-water-contact materials under chlorinated and non-chlorinated conditions.

Material	Non-Chlorinated Water	Chlorinated Water	Main Observation
M1	PET (20–100 µm) and ABS (20–100 µm)	PET (20–100 µm) dominant	Chlorination increased PET release and shifted the profile towards polyester-derived particles.
M2	PET (20–100 µm) dominant	PET (20–100 µm) and PA (20–300 µm) dominant	Highest diversity of released polymers and strongest chlorination effect.
M3	PE mainly 50–100 µm	PE mainly 50–100 µm	Particle composition reflects the original pipe material.
M4	Low concentrations, mainly PP and PET	Low concentrations, mainly PP and PET	Stable behavior and limited particle release.
M5	PVC predominantly 20–100 µm	PVC predominantly 20–100 µm	Release profile consistent with PVC-O composition.
M6	Very low particle abundance	Very low particle abundance	Lowest release potential among investigated materials.

**Table 3:** Relative contribution of drinking-water-contact materials to potential consumer exposure, expressed as estimated daily intake (EDI), annual estimated intake (AEI), and Relative Microplastic Release Index (RMRI).

Material	Σ*C_Tap_* (MPs/L)	EDI (MPs/d)	AEI (MPs/yr)	RMRI
	Non-Chlorinated Water			
M1	6.3	12.6	4599	0.20
M2	18.6	37.2	13,578	0.58
M3	14.2	28.4	10,366	0.44
M4	1.4	2.8	1022	0.04
M5	32.1	64.2	23,433	1.0
M6	0	0	0	0
	Chlorinated Water			
M1	22.9	45.8	16,717	0.33
M2	69.9	139.8	51,027	1.00
M3	3.9	7.8	2847	0.06
M4	5.4	10.8	3942	0.08
M5	17.3	34.6	12,629	0.25
M6	8	16	5840	0.11

**Table 4:** Physical, chemical, thermal, and functional characteristics of the materials employed in the investigated water distribution network, including thermoset resins, thermoplastic pipes, and cementitious protective linings.

ID	Material Type	Color/ Opacity	Material Family	Physical–Chemical Properties	Thermal Properties	Application	Functional Properties
M1	TP1887 Terephthalic Polyester Resin	Amber/ transparent	Thermoset polyester resin	Density 1.16 g/cm^3^; viscosity 300–400 cP; TS 75 MPa; FS 120 MPa; modulus 3500 MPa	HDT 75 °C; flash point 34 °C	GRP filament-wound pipes	High strength, chemical resistance, fiber wetting
M2	CE66 Isophthalic Polyester Resin	Clear/ transparent	Thermoset polyester resin	Density 1.10 g/cm^3^; TS 63 MPa; FS 125 MPa; impact 11 kJ/m^2^	HDT 85 °C; flash point 33 °C	GRP pipes/composites	Hydrolytic and chemical resistance
M3	HDPE PE100	Black/ opaque	HDPE	PE 97.5–98%; carbon black 2–2.5%; SG 0.95–0.97	Melting 125–135 °C; autoignition 350 °C	Pressure pipe manufacture	Ductility, corrosion resistance
M4	PE	Black/ opaque	Thermoplastic PE pipe	BS EN12201 potable-water -certified PE system [25]	Typical PE service temperatures	Water distribution pipelines	Flexible, leak-tight, corrosion-resistant
M5	PVC-O	Blue/ opaque	Oriented PVC	Density 1.37–1.43; tensile > 48 MPa; modulus 4000 MPa	Vicat ≥80 °C; low thermal conductivity	Water transmission pipes	Hydraulic efficiency
M6	Cementitious material	Gray/ opaque	Cementitious lining	Cement/sand/water 2:3:1; potable-water-compliant	Approved up to 65 °C	Internal DI pipe lining	Corrosion protection and water quality

**Abbreviations:** cP, centipoise; GRP, glass-reinforced plastic; HDPE, high-density polyethylene; HDT, heat deflection temperature; ID identification of material; MPa, megapascal; PE, polyethylene; PVC-O, oriented polyvinyl chloride; TS, tensile strength; FS, flexural strength; YS, yield strength; organic compounds; Vicat, vicat softening temperature.

**Table 5:** Dimensions, *S/V* ratio and conversion factor (CF) of the materials (M1-M6).

Parameter	Material					
	M1	M2	M3	M4	M5	M6
Length (mm)	145	145	397	400	118	93
Width (mm)	151	151	---	---	98	90
External diameter (mm)	---	---	50	50	---	---
Internal diameter (mm)	---	---	40	42	---	---
Height/Thickness (mm)	12	9	---	---	9	8
Recommended *S*/*V* ratio (dm^−1^)	5–40					5.0
Adopted *S*/*V* ratio (dm^−1^)	6.4	6.1	10.3	9.8	5.4	5.0
CF (d/dm)	5	5	10	10	10	10

## Data Availability

The data from materials in contact with drinking water are private due to the identification of materials and the applicant.

## Supplementary Materials

The following supporting information can be downloaded at: https://www.mdpi.com/article/10.3390/molecules31213960/s1, Table S1. Migration behavior of two materials, M1 and M2, with chlorinated water and non-chlorinated water (*n* = 2) expressed by number of MP particles of each polymer (MPs/L) across 1st, 2nd and 3rd migration (Migr.) cycles. Table S2. Migration behavior of two materials, M3 and M4, with chlorinated water and non-chlorinated water (*n* = 2) expressed by number of MP particles of each polymer (MPs/L) across 1st, 2nd and 3rd migration (Migr.) cycles. Table S3. Migration behavior of two materials, M5 and M6, with chlorinated water and non-chlorinated water (*n* = 2) expressed by number of MP particles of each polymer (MPs/L) across 1st, 2nd and 3rd migration (Migr.) cycles. Table S4. Size distribution of microplastics released during the third Migr. cycle from drinking-water-contact materials (M1-M6) under chlorinated (Ch) and non-chlorinated conditions (desmineralizated water, DW) expressed in an area equivalent diameter (AED).
