# Supplementary material for: Microplastic Release from Drinking Water Contact Materials: Material-Specific Responses to Chlorination and Implications for Consumer Exposure
Source: Molecules. 2026 Nov 3;31(21):3960. doi: 10.3390/molecules31213960 (PMC13764726; doi:10.3390/molecules31213960)
Supplement: Supplementary file 1 [file molecules-31-03960-s001.zip › molecules-4628873-supplementary.pdf]

Microplastic Release from Drinking Water Contact Materials: Material-Specific Responses to Chlorination and Implications for Consumer Exposure

Rodrigo D. M. Cordeiro[1], Ana Penetra [1], Rui N. Carneiro [1], Vitor V. Cardoso [1], and Cristina M. M. Almeida [2,*]

[1] Direção de Laboratórios, S.A.–EPAL, Empresa Portuguesa das Águas Livres, 1250-144 Lisboa, Portugal; rodrigo.cordeiro@adp.pt (R.D.M.C.); ana.penetra@adp.pt (A.P.); rui.carneiro@adp.pt (R.N.C.); vitor.cardoso@adp.pt (V.V.C.)

[2] iMed.UL, Faculdade de Farmácia, Universidade de Lisboa, 1649-003 Lisboa, Portugal

* Correspondence: calmeida@ff.ulisboa.pt

Supplementary Material

Table S1. Migration behavior of two materials, M1 and M2, with chlorinated water and non-chlorinated water (*n*=2) expressed by number of MP particles of each polymer (MPs/L) across 1st, 2nd and 3rd migration (Migr.) cycles.

MPs	M1						M2					
	Non-Chlorinated Water			Chlorinated Water			Non-Chlorinated Water			Chlorinated Water		
	1st Migr.	2nd Migr.	3rd Migr.	1st Migr.	2nd Migr.	3rd Migr.	1st Migr.	2nd Migr.	3rd Migr.	1st Migr.	2nd Migr.	3rd Migr.
EPDM	4	0	0	0	8	0	0	8	0	0	4	0
PVDF	0	0	0	0	0	0	4	0	0	0	56	60
PA	4	0	0	0	12	0	8	8	8	0	148	116
PC	0	0	0	0	0	0	0	0	0	0	0	0
PVC	0	0	0	4	0	0	0	0	0	0	0	4
PS	0	12	4	0	8	0	0	12	0	0	120	8
PE	0	0	0	0	0	0	0	0	0	0	120	12
PMMA	0	0	0	4	0	0	4	4	0	0	0	0
PP	0	0	0	4	0	4	8	4	4	0	60	28
PET	4	28	8	48	52	56	80	112	28	0	8	0
PTFE	0	0	0	0	0	0	0	0	0	0	68	28
PUR	0	0	0	0	0	0	0	0	0	0	0	0
ABS	4	12	12	44	20	28	36	16	28	0	0	0
∑MPs/L												
Total	16	52	24	104	100	88	140	164	68	0	584	256

Table S2. Migration behavior of two materials, M3 and M4, with chlorinated water and non-chlorinated water (*n*=2) expressed by number of MP particles of each polymer (MPs/L) across 1st, 2nd and 3rd migration (Migr.) cycles.

MPs	M3						M4					
	Non-Chlorinated Water			Chlorinated Water			Non-Chlorinated Water			Chlorinated Water		
	1st Migr.	2nd Migr.	3rd Migr.	1st Migr.	2nd Migr.	3rd Migr.	1st Migr.	2nd Migr.	3rd Migr.	1st Migr.	2nd Migr.	3rd Migr.
**EPDM**	0	0	0	0	0	0	12	0	0	0	0	0
**PVDF**	0	0	0	0	0	0	0	0	0	0	0	0
**PA**	0	4	0	0	0	4	8	0	0	8	0	0
**PC**	0	0	0	0	0	0	0	0	0	0	0	0
**PVC**	0	0	0	0	0	0	12	0	0	0	4	0
**PS**	0	8	4	0	0	0	0	0	0	0	0	0
**PE**	64	80	40	48	100	8	112	44	4	92	76	0
**PMMA**	0	0	0	0	0	0	0	0	0	0	0	0
**PP**	16	8	0	8	12	0	12	0	0	4	4	12
**PET**	8	16	0	64	16	0	8	0	0	16	8	4
**PTFE**	0	0	0	0	0	0	4	4	0	0	0	0
**PUR**	0	0	0	0	0	0	0	0	0	0	0	0
**ABS**	0	0	0	0	0	0	0	0	0	0	0	0
**∑MPs/L**												
**Total**	88	116	44	120	128	12	168	48	4	120	92	16

Table S3. Migration behavior of two materials, M5 and M6, with chlorinated water and non-chlorinated water (n=2) expressed by number of MP particles of each polymer (MPs/L) across 1st, 2nd and 3rd migration (Migr.) cycles.

MPs	M5						M6					
	Non-Chlorinated Water			Chlorinated Water			Non-Chlorinated Water			Chlorinated Water		
	1st Migr.	2nd Migr.	3rd Migr.	1st Migr.	2nd Migr.	3rd Migr.	1st Migr.	2nd Migr.	3rd Migr.	1st Migr.	2nd Migr.	3rd Migr.
**EPDM**	0	0	0	0	0	0	0	0	0	0	0	0
**PVDF**	0	0	0	0	0	0	0	0	0	0	0	0
**PA**	0	0	12	4	0	0	0	4	0	0	0	0
**PC**	0	0	0	0	4	0	0	0	0	0	0	0
**PVC**	20	20	20	56	36	28	0	4	0	0	0	0
**PS**	0	0	0	8	0	0	0	0	0	8	0	4
**PE**	0	0	0	0	0	0	12	0	0	8	4	0
**PMMA**	0	0	0	0	0	0	0	0	0	0	0	0
**PP**	0	10	10	0	0	0	12	4	0	0	0	8
**PET**	0	10	10	0	4	0	0	0	0	20	0	0
**PTFE**	0	0	0	0	0	0	4	0	0	0	0	0
**PUR**	0	0	0	0	0	0	0	0	0	0	0	0
**ABS**	0	0	0	0	0	0	0	0	0	0	0	0
**∑MPs/L**												
**Total**	20	40	52	68	44	28	28	12	0	36	4	12

Table S4. Size distribution of microplastics released during the third Migr. cycle from drinking-water-contact materials (M1-M6) under chlorinated (Ch) and non-chlorinated conditions (desmineralizated water, DW) expressed in an area equivalent diameter (AED).

MPs	AED (µm)	M1		M2		M3		M4		M5		M6	
		DW	Ch	DW	Ch	DW	Ch	DW	Ch	DW	Ch	DW	Ch
EPDM	20 - 50 µm	0	0	0	0	0	0	0	0	0	0	0	0
	50 - 100 µm	0	0	0	4	0	0	0	0	0	0	0	0
	100 - 300 µm	0	0	0	0	0	0	0	0	0	0	0	0
	> 300 µm	0	0	0	0	0	0	0	0	0	0	0	0
PVDF	20 - 50 µm	0	0	0	0	0	0	0	0	0	0	0	0
	50 - 100 µm	0	0	0	0	0	0	0	0	0	0	0	0
	100 - 300 µm	0	0	0	0	0	0	0	0	0	0	0	0
	> 300 µm	0	0	0	0	0	0	0	0	0	0	0	0
PA	20 - 50 µm	0	0	8	0	0	0	0	0	2	0	0	0
	50 - 100 µm	0	0	0	0	0	0	0	0	0	0	0	0
	100 - 300 µm	0	0	0	0	0	4	0	0	10	0	0	0
	> 300 µm	0	0	0	0	0	0	0	0	0	0	0	0
PC	20 - 50 µm	0	0	0	0	0	0	0	0	0	0	0	0
	50 - 100 µm	0	0	0	0	0	0	0	0	0	0	0	0
	100 - 300 µm	0	0	0	0	0	0	0	0	0	0	0	0

	**> 300 µm**	0	0	0	0	0	0	0	0	0	0	0	0
**PVC**	**20 - 50 µm**	0	0	0	0	0	0	0	0	10	16	0	0
	**50 - 100 µm**	0	0	0	0	0	0	0	0	10	12	0	0
	**100 - 300 µm**	0	0	0	0	0	0	0	0	0	0	0	0
	**> 300 µm**	0	0	0	0	0	0	0	0	0	0	0	0
**PS**	**20 - 50 µm**	0	0	0	4	4	0	0	0	0	0	0	4
	**50 - 100 µm**	4	0	0	4	0	0	0	0	0	0	0	0
	**100 - 300 µm**	0	0	0	0	0	0	0	0	0	0	0	0
	**> 300 µm**	0	0	0	0	0	0	0	0	0	0	0	0
**PE**	**20 - 50 µm**	0	0	0	0	0	0	0	0	0	0	0	0
	**50 - 100 µm**	0	0	0	0	32	0	0	0	6	0	0	0
	**100 - 300 µm**	0	0	0	0	8	8	4	0	0	0	0	0
	**> 300 µm**	0	0	0	0	0	0	0	0	0	0	0	0
**PP**	**20 - 50 µm**	0	0	4	0	0	0	0	0	0	0	0	8
	**50 - 100 µm**	0	4	0	12	0	0	0	12	0	0	0	0
	**100 - 300 µm**	0	0	0	0	0	0	0	0	10	0	0	0
	**> 300 µm**	0	0	0	0	0	0	0	0	0	0	0	0
**PET**	**20 - 50 µm**	4	20	12	20	0	0	0	0	10	0	0	0
	**50 - 100 µm**	4	28	12	12	0	0	0	0	0	0	0	0
	**100 - 300 µm**	0	8	4	0	0	0	0	0	0	0	0	0

	**> 300 µm**	0	0	0	0	0	0	0	4	0	0	0	0
**PU**	**20 - 50 µm**	0	0	0	0	0	0	0	0	0	0	0	0
	**50 - 100 µm**	0	0	0	0	0	0	0	0	0	0	0	0
	**100 - 300 µm**	0	0	0	0	0	0	0	0	0	0	0	0
	**> 300 µm**	0	0	0	0	0	0	0	0	0	0	0	0
**ABS**	**20 - 50 µm**	0	0	0	0	0	0	0	0	0	0	0	0
	**50 - 100 µm**	0	20	24	4	0	0	0	0	0	0	0	0
	**100 - 300 µm**	12	8	4	0	0	0	0	0	0	0	0	0
	**> 300 µm**	0	0	0	0	0	0	0	0	0	0	0	0
